# Lysine acetylation of *Escherichia coli* lactate dehydrogenase regulates enzyme activity and lactate synthesis

**DOI:** 10.3389/fbioe.2022.966062

**Published:** 2022-08-16

**Authors:** Min Liu, Meitong Huo, Changshui Liu, Likun Guo, Yamei Ding, Qingjun Ma, Qingsheng Qi, Mo Xian, Guang Zhao

**Affiliations:** ^1^ State Key Laboratory of Microbial Technology, Shandong University, Qingdao, China; ^2^ Institute of Oceanology, Chinese Academy of Sciences, Qingdao, China; ^3^ CAS Key Laboratory of Biobased Materials, Qingdao Institute of Bioenergy and Bioprocess Technology, Chinese Academy of Sciences, Qingdao, China

**Keywords:** lysine acetylation, lactate dehydrogenase A, enzyme activity, metabolic engineering, lactate synthesis

## Abstract

As an evolutionarily conserved posttranslational modification, protein lysine acetylation plays important roles in many physiological and metabolic processes. However, there are few reports about the applications of lysine acetylation in metabolic regulations. Lactate is a main byproduct in microbial fermentation, and itself also an important bulk chemical with considerable commercial values in many fields. Lactate dehydrogenase (LdhA) is the key enzyme catalyzing lactate synthesis from pyruvate. Here, we reported that *Escherichia coli* LdhA can be acetylated and the acetylated lysine sites were identified by mass spectrometry. The effects and regulatory mechanisms of acetylated sites on LdhA activity were characterized. Finally, lysine acetylation was successfully used to regulate the lactate synthesis. LdhA (K9R) mutant overexpressed strain improved the lactate titer and glucose conversion efficiency by 1.74 folds than that of wild-type LdhA overexpressed strain. LdhA (K154Q-K248Q) mutant can inhibit lactate accumulation and improve 3HP production. Our study established a paradigm for lysine acetylation in lactate synthesis regulation and suggested that lysine acetylation may be a promising strategy to improve the target production and conversion efficiency in microbial synthesis. The application of lysine acetylation in regulating lactate synthesis also provides a reference for the treatment of lactate-related diseases.

## Introduction

In order to adapt a changing environment, cells undergo complex regulations at different levels. Among them, posttranslational modification (PTM) can covalently modify amino acid by biochemical mechanism to make the protein structure more complex and the regulatory effect more precise (Liu et al., 2021). Protein lysine acetylation is a highly conserved PTM from bacteria to higher animals (Hentchel and Escalante-Semerena, 2015; VanDrisse and Escalante-Semerena, 2019). Acetylation modification induces charge change of lysine from +1 to 0 and adds a bigger side chain of acetyl group. Lysine acetylation contains enzymatic and nonenzymatic mechanisms. In enzymatic acetylation, lysine acetyltransferase (KAT) catalyzes the transfer of an acetyl group from acetyl coenzyme A (Acetyl-CoA) to target protein lysine residues. In nonenzymatic acetylation, an acetyl group from acetyl phosphate (AcP) direct transfers to protein lysine residues. AcP- mediated nonenzymatic acetylation is the primary mechanism of *E. coli* (Liu et al., 2021).

Previously, researches on protein acetylation mainly focused on histone modification for regulating gene transcription in eukaryotes (Verdin and Ott, 2015). In recent decades, many bacterial acetylated proteins were constantly discovered with the development of high-affinity immune separation and nano-HPLC/MS/MS (Yu et al., 2008; Zhang et al., 2009; Zhang et al., 2013; Kuhn et al., 2014). Lysine acetylation is abundant in bacteria, affecting the cellular physiology and metabolism (Wang, 2010; Liu et al., 2021). Thus, lysine acetylation may become a promising tool in metabolic engineering. However, the applications of lysine acetylation in metabolic regulations are rarely reported. Furthermore, it remains unclear how lysine acetylation affects the cellular physiology and metabolism.

NAD-dependent lactate dehydrogenase (LdhA) is a conserved protein presented in many species, specific for lactate biosynthesis from pyruvate. In human, lactate has been proved to play an important role in pathogenesis of cancer and diabetes (Feng et al., 2018; Lin et al., 2022), and lysine acetylation regulates the activity of human LdhA, further affecting cell migration and proliferation in pancreatic cancer (Zhao et al., 2013). On the other hand, lactate itself is an important bulk chemical, widely used in the fields of cosmetics, herbicides and pharmaceutical (Feng et al., 2014; Choi et al., 2019). Lactate is also used as the precursor for producing the bioplastic polymers of polylactic acid (PLA). At present, the majority of lactate is produced by microbial fermentation because of some outstanding advantages over the chemical synthesis, like the environmental protection, low cost and low energy consumption (Abdel-Rahman et al., 2013; Juturu and Wu, 2016). In addition, lactate accumulation is also a main problem in bioproduction process of other chemicals, which is caused by the intracellular accumulation of pyruvate and NADH, as a consequence of the imbalance between rapid glucose catabolism and the limited respiratory capacity of microbe (Eiteman and Altman, 2006; Bernal et al., 2016). Traditionally, *ldhA* gene was deleted in engineered microorganism to repress the lactate production, that brought about some negative issues. So, regulation of lactate production is an enduring research topic in metabolic engineering.

*Escherichia coli* is one of the most widely used host in microbial fermentation, employed to synthesize a large amount of chemicals including lactate, but it still remains unknown whether the activity of *E. coli* LdhA is affected by lysine acetylation. In this study, we found lysine acetylation can affect LdhA function in *E. coli*. We identified the acetylated sites and explored the mechanism of LdhA acetylation on enzyme activity. Finally, we established a paradigm of lactate synthesis regulation by lysine acetylation.

## Materials and methods

### Plasmids and strains construction

Primers used in this study were listed in Supplementary Table S1, all plasmids and strains used in this study were listed in Table 1. *E. coli* DH5α was used as the host for plasmid construction, and *E. coli* BL21 (DE3) was used as the host for protein expression and target production. Polymerase chain reaction (PCR) combined with restriction enzyme digest were used to plasmid construction. Site-directed mutagenesis of *ldhA* was carried out according to the Stratagene protocol. The chromosomal genes of *E. coli* BL21 (DE3) were knocked out *via* P_1_ vir-mediated transduction as previously described (Moore, 2011). The donor strains were purchased from the Keio collection (Baba et al., 2006). The *ldhA* (K154Q-248Q) replacement *in situ* was carried out by suicide plasmid pRE112-mediated homologous recombination (Edwards et al., 1998). The recombinant plasmids were transformed to their corresponding hosts for protein expression and fermentation.

**TABLE 1:** Plasmids and strains used in this study.

Plasmids and strains	Relevant properties	Source
Plasmids		
pACYCDuet1	*Cm* ^r^ *oriP*_*15A*_ *lacI* ^*q*^ P_*T7*_	Novagen
pETDuet1	Amp^r^ oriP_BR322_ lacIq P_T7_	Novagen
pCP20	reppSC101^ts^ Ap^R^ Cm^R^ cI857 λP_R_ FLP	CGSC^a^
pRE112	oriT oriV sacB Cm^R^	Dr. Roy Curtiss III
pACYCDuet1-*acc*	rep_p15A_ Cm^R^ lacI P_T7_ *accA*_PT7_ *accD*_PT7_ *accBC*	Liu et al. (2016)
pETDuet1-*mcr*	rep_pBR322_ Amp^R^ lacI P_lac,p2-51_ -*mcr*1-549 P_T7_ *mcr*550-1,219(N940 V K1106W S1114R)	Liu et al. (2016)
pETDuet1-*ldhA*	Amp^r^ oriP_BR322_ lacIq P_T7_ ldhA	This study
Strains		
*E. coli* DH5α	F^−^ *supE*44 Δ*lacU*169 (*ϕ*80 *lacZ* Δ*M15*) *hsdR*17 *recA*1 *endA*1 *gyrA*96 *thi*-1 *relA*1	Invitrogen
*E. coli* BL21 (DE3)	F^−^ *ompT gal dcm lon hsdSB* (rB^−^ mB^−^) λ(DE3)	Invitrogen
*E. coli* χ7213	*thi*-1 *thr*-1 *leu*B6 *gln*V44 *fhu*A21 *lac*Y1 *rec*A1 *RP4*-2-Tc:Mu λ*pir* Δ*asd*A4 Δ*zhf*-2:*Tn*10	Dr. Roy Curtiss III
Q3685	*E. coli* BL21 (DE3)/pETDuet1-*ldhA*	This study
Q3749	*E. coli* BL21 (DE3)/pETDuet1-*ldhA* (K9R)	This study
Q3750	*E. coli* BL21 (DE3)/pETDuet1-*ldhA* (K9Q)	This study
Q3686	*E. coli* BL21 (DE3)/pETDuet1-*ldhA* (K70R)	This study
Q3687	*E. coli* BL21 (DE3)/pETDuet1-*ldhA* (K70Q)	This study
Q3688	*E. coli* BL21 (DE3)/pETDuet1-*ldhA* (K154R)	This study
Q3689	*E. coli* BL21 (DE3)/pETDuet1-*ldhA* (K154Q)	This study
Q3690	*E. coli* BL21 (DE3)/pETDuet1-*ldhA* (K248R)	This study
Q3691	*E. coli* BL21 (DE3)/pETDuet1-*ldhA* (K248Q)	This study
Q3714	*E. coli* BL21 (DE3)/pETDuet1-*ldhA* (K70Q-K154Q)	This study
Q3715	*E. coli* BL21 (DE3)/pETDuet1-*ldhA* (K154Q-K248Q)	This study
Q3719	*E. coli* BL21 (DE3)/pETDuet1-*ldhA* (D279E)	This study
Q3720	*E. coli* BL21 (DE3)/pETDuet1-*ldhA* (D279N)	This study
Q3786	*E. coli* BL21 (DE3)/pETDuet1-*ldhA* (E269D)	This study
Q3787	*E. coli* BL21 (DE3)/pETDuet1-*ldhA* (E269N)	This study
JW1375	BW25113 *ΔldhA::Km* ^*R*^	Keio collection
Q2326	*E. coli* BL21 (DE3) Δ*ldhA*	This study
Q3784	*E. coli* BL21 (DE3) Δ*ldhA*/pETDuet1-*ldhA*	This study
Q3785	*E. coli* BL21 (DE3) Δ*ldhA*/pETDuet1-*ldhA* (K9R)	This study
Q3790	*E. coli* BL21 (DE3) *ldhA*: *ldhA* (K154Q-K248Q)	This study
Q2191	*E. coli* BL21 (DE3)*/*pACYCDuet1-*acc/*pETDuet1-*mcr*	This study
Q 3824	Q2326*/*pACYCDuet1-*acc/*pETDuet1-*mcr*	This study
Q 3825	Q3790*/*pACYCDuet1-*acc/*pETDuet1-*mcr*	This study

a The Coli Genetic Stock Center at Yale University.

### Identification of acetylated lysine residues by mass spectrometry

The purified LdhA cultured in Luria–Bertani (LB) with 2% glucose was detected by 12% SDS-PAGE, the excised LdhA band was digested with trypsin by a standard in-gel digestion protocol. The trypsin digested product was desalted by Ziptip C18 chromatographic column and redissolved by 0.1% trifluoroacetic acid. Peptides were separated on a nanoViper C18 silica column Acclaim PepMap RSLC (75 μm × 25 cm, 2 μm, Thermo, United States) with mobile phase system of solvent A (0.1% formic acid) and B (80% ACN and 0.1% formic acid) at a flow rate of 300 nL/min, and analyzed by nano system (Thermo Scientific, EASY-nLC, United States) coupled with a 1,000,000 FWHM high-resolution Nano Orbitrap Fusion Lumos Tribrid Mass Spectrometer system (Thermo Scientific, United States). Mass spectrometric data processing used the Proteome Discoverer software 2.3 (Thermo Scientific, United States).

### Protein expression and purification

*E. coli* cells were grown overnight at 37°C in LB medium with appropriate antibiotics. The culture was diluted 1:50 into fresh LB medium with 2% glucose and incubated under the same condition. When the OD_600_ of culture reached about 0.8, 100 μM isopropyl-β-d-thiogalactopyranoside (IPTG) was added for T_7_ promoter induction and growth was continued for 18 h at 30°C. Cells were collected by centrifugation and resuspended in phosphate buffer saline buffer (pH 7.5), and subjected to high pressure. The cell lysates were centrifuged and purified using Ni-NTA His·Bind Column (Novagen) according to the manufacturer’s instruction. The purified proteins were used for detecting the lysine acetylation and enzyme activity.

### Western blotting

The concentrations of purified protein samples were determined by A280 absorption and fractionated on a 12% SDS-PAGE gel. Then, the protein samples were transferred to polyvinylidene difluoride (PVDF) membranes for 1.5 h at 15 V. The membrane was blocked at room temperature for 1 h using quick block western reagent (Beyotime, China). Acetyl lysine mouse monoclonal antibody (EasyBio, China, 1:2000) was used as the primary antibody and incubated overnight at 4°C, then Goat horseradish peroxidase-conjugated anti-mouse antibody diluted in PBST (EasyBio, China, 1:10,000) was used as the secondary antibody and incubated for 1 h. After washing three times with PBST, an enhanced chemiluminescence (ECL) system was used for signal detection according to the manufacturer’s instructions.

### LdhA activity assay

The reaction mixture contained 100 mM phosphate buffer (pH 7.5), 1 mM NADH, 1 mM pyruvate, and 40 nm purified protein. The specific activity was assayed by measuring the change in absorbance at 340 nm resulting from NADH oxidation using multimode microplate reader (Spark, Tecan).

### Shake-flask fermentation

The D-lactate production of Q3784 and Q3785 in shake flask cultivation were performed as previously described methods (Feng et al., 2014). Strains were cultivated in a 250 ml flask containing 100 ml medium at 37°C with 100 rpm. When OD_600_ reached about 0.8, 100 µM IPTG was added and further incubated 24 h at 30°C. The pH was maintained at 7.0 with ammonia every 12 h. The 3HP production of the Q2191, Q3824, and Q3825 strains in shake flask cultivation were referred in (Liu et al., 2016). The strains were grown overnight at 37°C in LB broth and then 1:50 diluted into 250 ml Erlenmeyer flasks with 50 ml minimal medium. When OD_600_ of the culture reached about 0.6, IPTG was added to a final concentration of 100 µM and further incubated at 30°C for 48 h. The OD_600_, the concentrations of residual glucose and intermediates were measured during the whole fermentation course.

### Analytic methods

Cell growth was assayed by measuring optical density of the culture at 600 nm using a spectrophotometer (U-2900; Hitachi). The residual glucose concentration was detected by an SBA-40ES biological sensing analyzer (Institute of Biology, Shandong Academy of sciences, China). Metabolites were analyzed by an Agilent 1,260 Infinity series HPLC system equipped with an HPX-87H column (Bio-Rad, Hercules, CA) (300 mm × 7.8 mm). All samples were filtered through 0.22 μm syringe filter. 5 mM H_2_SO4 was used as the eluent at 0.5 ml/min. The oven temperature was maintained at 40°C. Concentrations of metabolites were calculated according to the standard curves.

## Results and discussion

### Identification of acetylated LdhA sties

**FIGURE 1:**
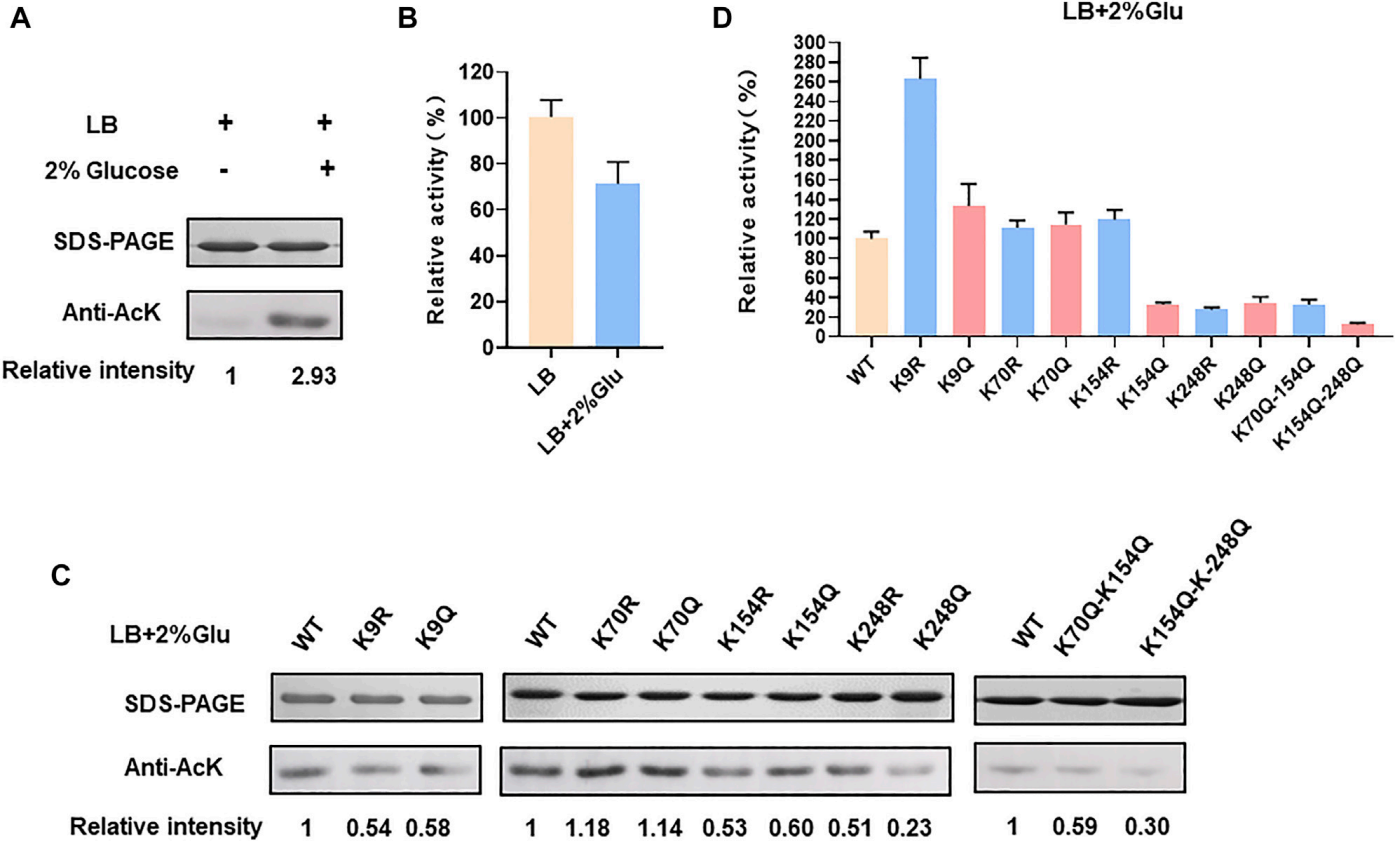
LdhA acetylation and enzyme activity in LB with 2% glucose cultivated condition. **(A)** SDS-PAGE and western blot analysis of LdhA cultivated in different conditions. **(B)** LdhA activity in cells grown in LB broth with and without 2% glucose supplementation. **(C)** SDS-PAGE and western blot analysis of LdhA variants with lysine substitution. **(D)** The enzyme activities of LdhA variants with lysine substitution.

In order to verify whether *E. coli* LdhA was acetylated, we firstly performed a western blot of LdhA cultivated in LB medium using an acetyl lysine monoclonal antibody. Though lysine acetylation of *E. coli* LdhA was detected by western blot, the acetylation level was weak in LB cultivated condition as shown in Figure 1A. Some previous studies have reported that different carbon source affects the acetylation status and glucose can improve the protein acetylation (Wang et al., 2010; Schilling et al., 2019). Different carbon source and concentration can affect the intracellular AcP level *via* acetate metabolism pathway, regulating protein acetylation level by AcP-mediated nonenzymatic mechanism (Schilling et al., 2019). Then, the lysine acetylation of LdhA cultivated in LB medium supplemented with 2% glucose were detected. Consistently with the previous reports, supplementation of glucose significantly improved the acetylation level of LdhA protein by 2.93-fold (Figure 1A).

Additionally, wild-type LdhA activity in *E. coli* cells grown with the presence and absence of 2% glucose were compared. LdhA activity decreased to about 71% with supplementation of 2% glucose (Figure 1B). Due to the need of positive charge of lysine residue in some interactions, improved acetylation level by 2% glucose supplementation reduced the overall activity of LdhA. For identifying more acetylated peptides, LdhA protein was purified from cells grown with 2% glucose, and analyzed by mass spectrometry. Four acetylated sites of K9, K70, K154, and K248 were detected, and all the acetylated LdhA peptides were presented in Table 2.

**TABLE 2:** Identification of acetylated LdhA peptides by mass spectrometry.

Site	Sequence
K9	LAVYSTK(Ac)QYDK
K70	HGVK(Ac)YIALR
K154	TAGVIGTGK(Ac)IGVAMLR
K248	IDSQAAIEALK(Ac)NQKIGSLGMD

### Effect of acetylated sites on LdhA activity

To avoid biased selection, we studied the effects of all the identified acetylated sites on LdhA activity. Each of these four lysine residues was substituted to arginine (R) and glutamine (Q) in which the substitution of arginine keeps the positive charge and avoids acetylation, whereas glutamine substitution neutralizes the positive charge and mimics the structure of acetylated lysine. Then all these mutants cultivated in LB with 2% glucose were characterized by western bolt and activity assay. For K70 mutants, neither acetylation level nor enzyme activity changed as compared to wild-type LdhA. In contrast, the acetylation levels of all other mutants decreased to varying degrees (Figure 1C), and the picture of enzyme activity change of these mutants was more complex. Specifically, the lysine acetylation level of K9 mutants, K154 mutants and K248R decreased to 0.5–0.6 folds, and the lysine acetylation level of K248Q decreased to 0.23-fold (Figure 1C). The enzyme activities of K154Q, K248R, and K248Q mutants decreased to about 30% of wild-type LdhA, while K9R mutation increased LdhA activity by 2.5 times (Figure 1D). K154R and K9Q mutations showed no obvious difference as compared to the LdhA activity, although they did lower the acetylation status of the LdhA protein (Figure 1D). Considering all these results, we speculated that K70 may be located at the non-core region of the structure and lysine acetylation did not affect the LdhA activity. While, K154 and K248 may be involved in binding the substrate and cofactor or maintaining the spatial conformation. Acetylation modification changes the charge status and adds an acetyl group to lysine residue, which may sterically block the catalytic pockets and affect the LdhA activity. Therefore, structural analysis of LdhA will help to reveal the regulation mechanism of acetylated lysine sites on enzyme activity.

Furthermore, two double mutants, K70Q-K154Q and K154Q-K248Q, were constructed and analyzed. The acetylation level and enzyme activity of K70Q-K154Q were similar to those of K154Q (Figures 1C,D), and this phenomenon is consistent with above result that K70Q cannot function on LdhA acetylation and enzyme activity. Though the acetylation level of K154Q-K248Q was similar as the single mutant K248Q, its activity was decreased to 12.6% (Figure 1D). In summary, each individual acetylated lysine residue has different effect on LdhA activity that may depend on the spatial location of each acetylated lysine. In addition, mutation of a single specific lysine or a combination of several specific lysines may achieve a desirable effect in metabolic regulation.

### Mechanism of acetylation regulating LdhA enzyme activity

Previous studies found that *E. coli* LdhA (EcLdhA) functions as a homotetramer with allosteric property, and pyruvate binding activates the enzyme with a potential conformational change, displaying the positive cooperativity among the monomers (Tarmy and Kaplan, 1968a; Tarmy and Kaplan, 1968b; Furukawa et al., 2014). Structural analysis showed each monomer of EcLdhA consists of a catalytic domain and an NAD-binding domain connected by two linkers (Furukawa et al., 2018). As known, EcLdhA belongs to the D-isomer-specific 2-hydroxyacid dehydrogenase family, in which the homolog members adopt a closed conformation when the catalytic domains bind substrates or the analogs (Taguchi and Ohta, 1991; Razeto et al., 2002; Furukawa et al., 2018). However, the complex structure of EcLdhA with pyruvate and NADH has not been reported, resulting in a limitation for our structural observation. To figure out how acetylation of each lysine residue regulates LdhA activity, it is needed to gain the complex structure of EcLdhA by homology modelling. The D-lactate dehydrogenases from *Pseudomonas aeruginosa* (PaLdhA) and *Fusobacterium nucleatum* (FnLdhA) share a high sequence identity of 54% and 47% with EcLdhA, respectively, and they both possess the ternary structures with a pyruvate analog and NADH (Furukawa et al., 2018). In contrast to PaLdhA, FnLdhA shares more similarity in the quaternary structure with EcLdhA, and they both exhibits a positive cooperativity of substrate binding compared to the negative cooperativity in the PaLdhA (Furukawa et al., 2014; Furukawa et al., 2018). Therefore, the FnLdhA ternary structure (PDB: 5Z21) was finally used as the template to simulate the complex structure of EcLdhA by Swiss-Model (Waterhouse et al., 2018).

**FIGURE 2:**
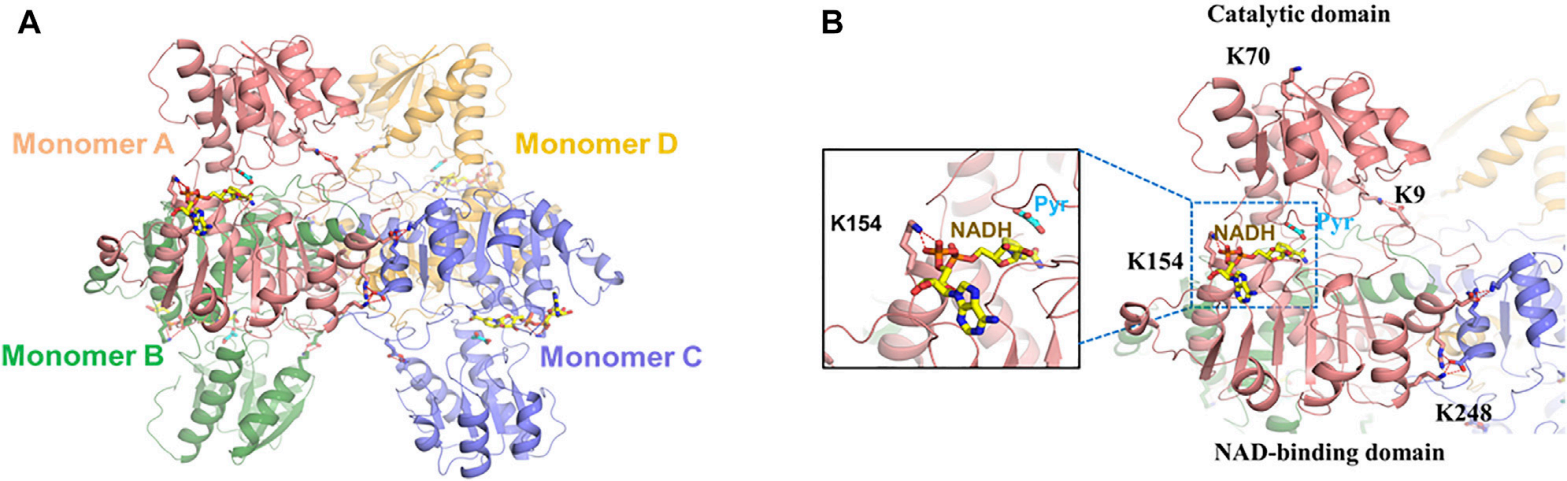
The 3D modelled structure of EcLdhA. **(A)** Quaternary structures of LdhA. **(B)** Location of the LdhA acetylated lysine sites, K70 is located at the distal region of the catalytic domain, K154 is responsible for binding the cofactor of NADH, K9 and K248 form the salt bridges with acidic amino acids, Pyr: pyruvate.

As shown in Figure 2A, EcLdhA presents a homotetrameric structure with each monomer in the closed state, and pyruvate and NADH are located at the catalytic domain and the NAD-binding domain, respectively (Figure 2B). According to this 3D modelled structure (Figure 2B), K70 is located at the distal region of the catalytic domain and has less impact on LdhA function, which is the reason why K70 substitutions did not change LdhA activity (Figure 2B). In contrast, K154 is responsible for binding the phosphate group of NADH, and replacement of this lysine by glutamine would impair this interaction, leading to a dramatic decrease of enzyme activity.

**FIGURE 3:**
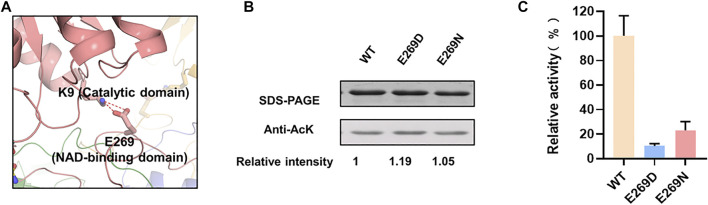
The mechanism of K9 acetylation on enzyme activity. **(A)** The salt bridge of K9-E269 in monomer. **(B)** The acetylation levels of E269 mutants. **(C)** The enzyme activities of E269 mutants.

K9 located at the catalytic domain may play an important role in maintaining the intramonomer interaction by forming a salt bridge with E269 located in NAD-binding domain (Figure 3A). To verify whether this salt bridge affects the LdhA activity, E269 was substituted to negative charged aspartic acid and neutral charged asparagine, respectively. As expected, E269 mutations didn’t change the overall acetylation status of LdhA (Figure 3B). The enzyme activities of E269D and E269N mutants were decreased to 10.3% and 23.2% (Figure 3C), which is probably due that improper charge and side chain length of position 269 residue blocked the formation of salt bridge with K9. It is worth mentioned that the activity of K9R mutant was increased by 2.5 times (Figure 1D), and it was assumed this activity improvement could be caused by two reasons. On one hand, substitution of lysine by arginine avoids acetylation of this residue; on the other hand, the bifurcated guanidine group of arginine is supposed to form stronger interactions with the carboxyl group of E269, maintaining the closed conformation favorably for substrate binding and improving the enzyme activity of LdhA.

**FIGURE 4:**
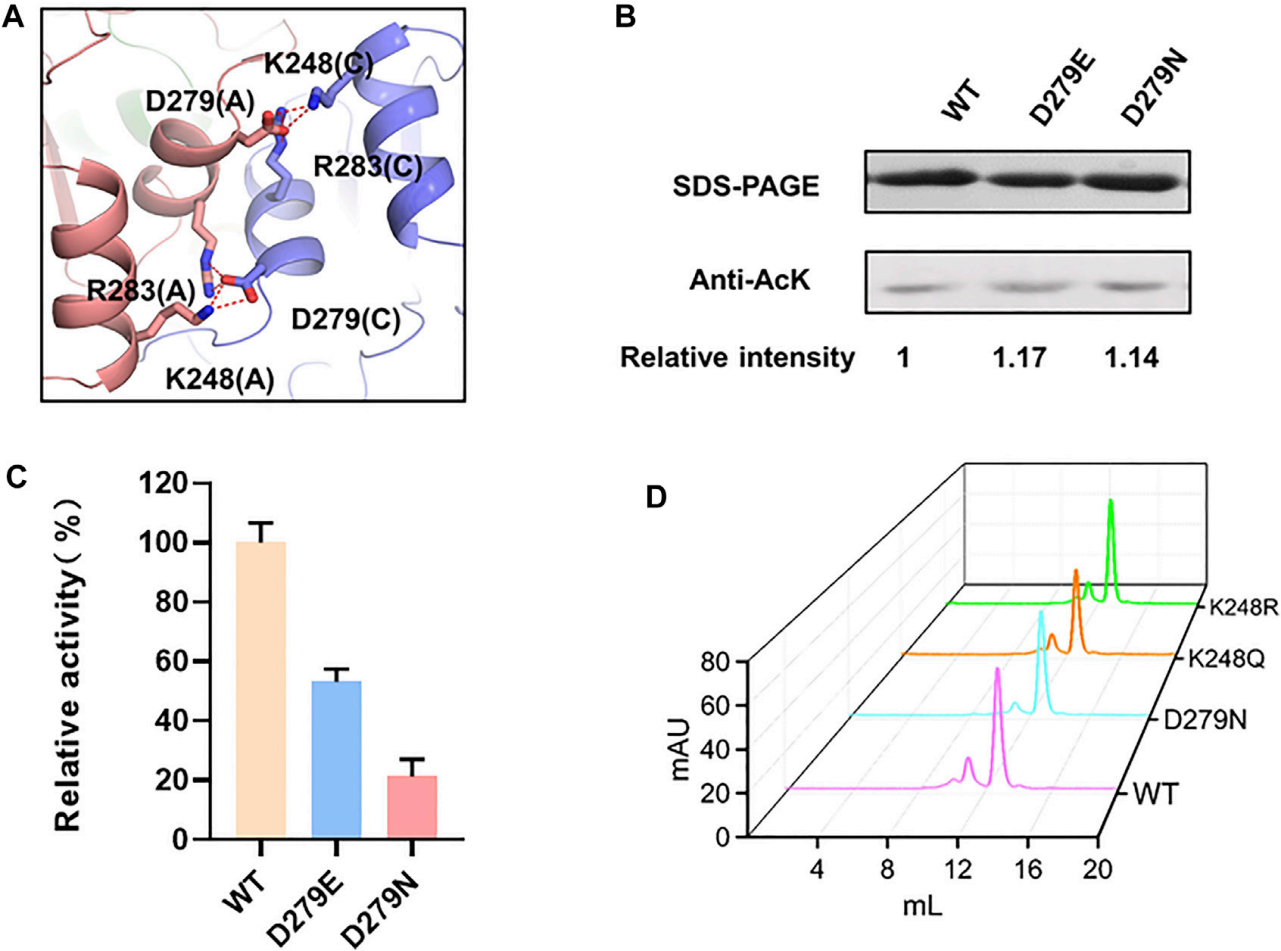
The mechanism of K248 acetylation on enzyme activity. **(A)** The intermonomer salt bridge of K248-D279. **(B)** The acetylation levels of D279 mutants. **(C)** The enzyme activities of D279 mutants. **(D)** Size exclusion chromatograph of LdhA mutants about the K248-D279 salt bridge.

Unlike the intramonomer salt bridge between K9-E269, K248 located at the NAD-binding domain forms a salt bridge with D279 from an adjacent monomer, contributing to the intermonomer interaction (Figure 4A). Similar to E269, D279 was substituted to glutamic acid and asparagine, leading to unchanged acetylation level and significantly reduced activity of LdhA (Figures 4B,C). To be noted, differently from the K9R mutant that may reinforce salt bridges for improved activity, K248R reduced LdhA activity to 27.5% (Figure 1D). Combined with the changed activity of the D279 mutations, it was confirmed that a certain impact on the intermonomer salt bridges between K248 and D279 results in the decreased activity of LdhA, and this impact should be further involved in an interfacial change which may alter the inherent allosteric effect of the tetramer or even cause tetramer dissociation. To test this hypothesis, the wild-type LdhA protein and K248R, K248Q, and D279N mutants were subjected to gel filtration analysis. The retention volumes of the highest peaks of these mutants were same as that of the wild type (Figure 4D), proving the presence of LdhA tetramer and that only preventing formation of the salt bridge between K248-D279 is not sufficient to destroy tetrameric state of LdhA. Hence, the salt bridges between K248 and D279 should be strictly required for the allosteric property of the tetramer, and the substitution by arginine may not achieve allosteric regulation to some extent. Sequence alignment further showed K248 is highly conserved in diverse bacterial species (Supplementary Figure S1), suggesting K248 should be a key residue to form the intermonomer interface and thus could be utilized as a target for regulating LdhA activity in bacteria.

**FIGURE 5:**
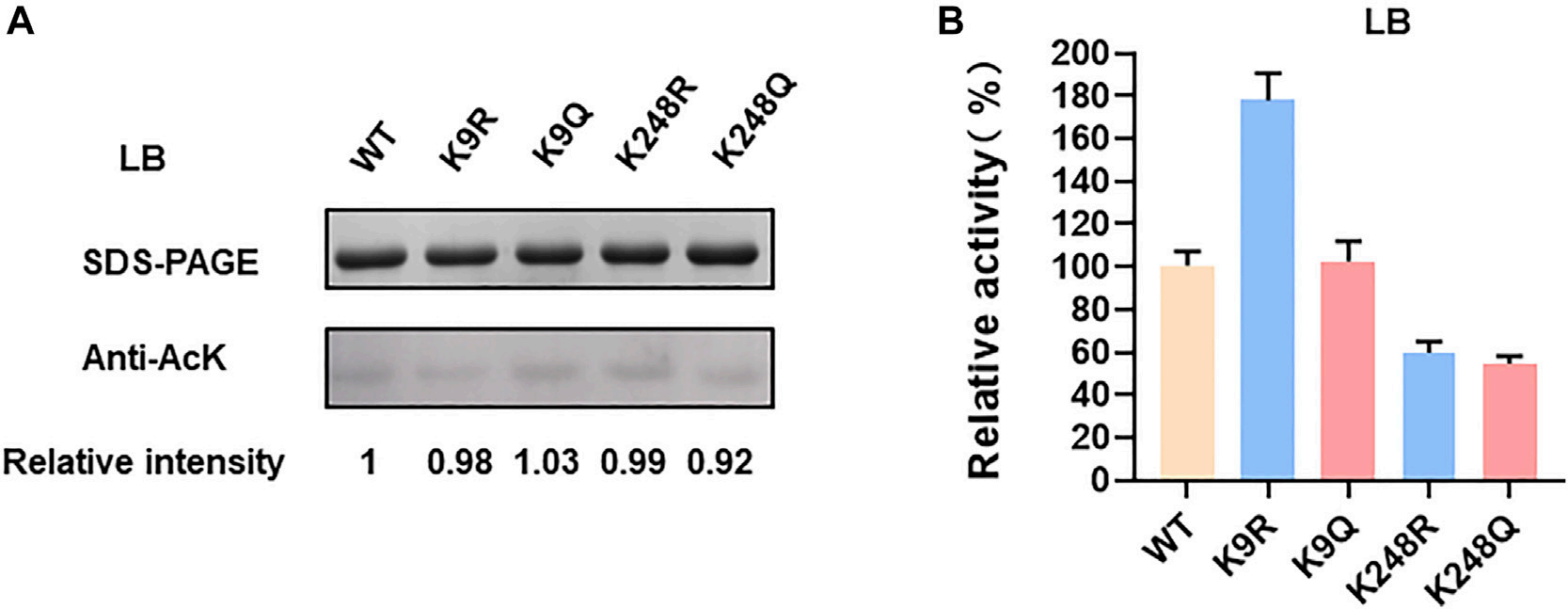
LdhA acetylation and enzyme activity in LB cultivated condition. **(A)** SDS-PAGE and western blot analysis of LdhA variants with lysine substitution. **(B)** The enzyme activities of LdhA variants with lysine substitution.

K9 and K248 are not the catalytic key sites, but they both help for keeping LdhA conformation by forming salt bridges with acidic amino acids. (Figure 2B). However, the mechanisms of K9 and K248 on LdhA conformation are different, which lead to different effects of lysine acetylation on enzyme activity. In order to investigate the effect of key lysine site mutation on enzyme activity, the acetylation status and enzyme activity of K9 and K248 mutants cultivated in LB medium with and without 2% glucose were compared. The acetylation levels of LdhA and mutants in LB medium were very low as compared to that in LB medium with 2% glucose (Figure 1A and Figure 5A). The enzyme activities of K248R and K248Q mutants in LB cultivated condition decreased to about 50% of wild-type LdhA, while K9R mutation increased LdhA activity by 1.8 times (Figure 5B). Due to the very low acetylation level, we concluded that the change of LdhA activity in LB cultivated condition is mainly resulted from the mutation of lysine residues. It is worth noting that mutation of lysine residue mimics the function of acetylation modification. The substitutions of lysine residue change the side chain size and charge. Therefore, when K9 and K248 mutants cultivated in LB +2% glucose medium, the improved acetylation modification further expanded the degree of variation. K248 mutants decreased LdhA activity to about 30% of wild-type LdhA and K9R mutant increased LdhA activity by 2.5 times (Figures 1B,C). Therefore, the effect of lysine acetylation on enzyme activity is depend on the contribution of positive charge and side chain size of lysine on structure. The spatial location of each lysine and acetylation modification work together to regulate the enzyme activity.

In conclusion, the mechanisms of acetylation of each lysine regulating LdhA activity are different, which mainly depends on the interaction modes of these residues. K9 and K248 form the intramonomer and intermonomer salt bridges with acidic amino acids, while K154 binds the phosphate group of NADH. All these interactions need the positive charge of lysine. Therefore, this study provided a new perspective for rationally regulation of enzyme activity by lysine acetylation modification.

### K9R mutant improves lactate production

**FIGURE 6:**
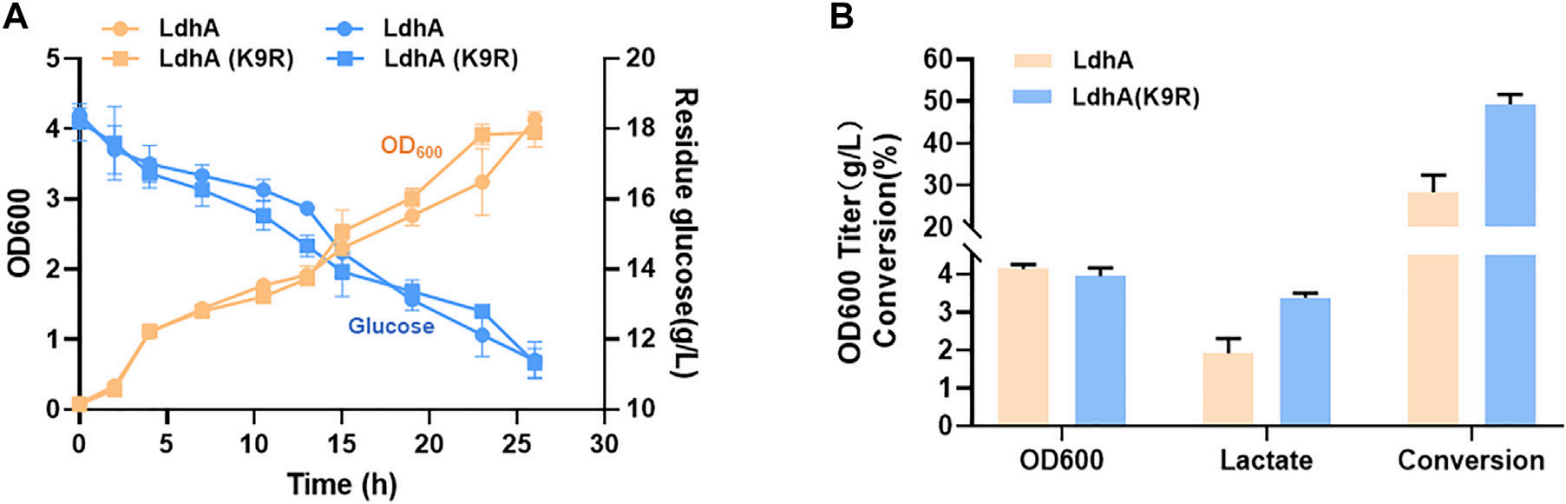
K9R mutant improves lactate production. **(A)** The cell growth and glucose consumption of wild LdhA and K9R mutant overexpressed strains. The yellow line represents the cell growth, the blue line represents the glucose consumption. **(B)** The biomass and metabolic profiles of wild LdhA and K9R mutant overexpressed strains in lactate fermentation.

Lactate is an important bulk chemical widely used in many fields. The strategies for improving lactate production in microbial fermentation mainly by inhibiting the byproducts accumulation, overexpressing *ldhA* gene of lactate synthetic pathway and optimizing fermentation conditions in some previous studies (Abdel-Rahman et al., 2013; Feng et al., 2014; Juturu and Wu, 2016; Feng et al., 2017). Elevating the LdhA activity by posttranslational modification may further improve the lactate production. According to the above results, LdhA (K9R) mutant significantly improved the activity of LdhA. Thus, the genes of *ldhA* and *ldhA* (K9R) mutant were respectively overexpressed in *E. coli* to produce lactate. The cell growth and glucose consumption of the two strains had no obvious difference (Figure 6A). The OD600 of wild LdhA and K9R mutant overexpressed strains were 4.13 and 3.95 respectively after 24 h fermentation. The lactate titer of LdhA (K9R) overexpressed strain was 3.38 g/L, much higher than that produced in wild LdhA overexpressed strain of 1.91 g/L. The conversion efficiency of K9R mutation was 49.23%, which was improved by 1.74 folds as compared to wild LdhA overexpressed strain of 28.37% (Figure 6B).

According to these results, LdhA (K9R) mutant can effectively improve the lactate production and glucose conversion efficiency by elevating enzyme activity. Therefore, the combination of lysine acetylation regulation and the traditional engineering strategies will achieve the more efficient synthesis of lactate and promote the industrial applications.

### K154Q-K248Q mutant improves 3HP production by inhibiting lactate accumulation

**FIGURE 7:**
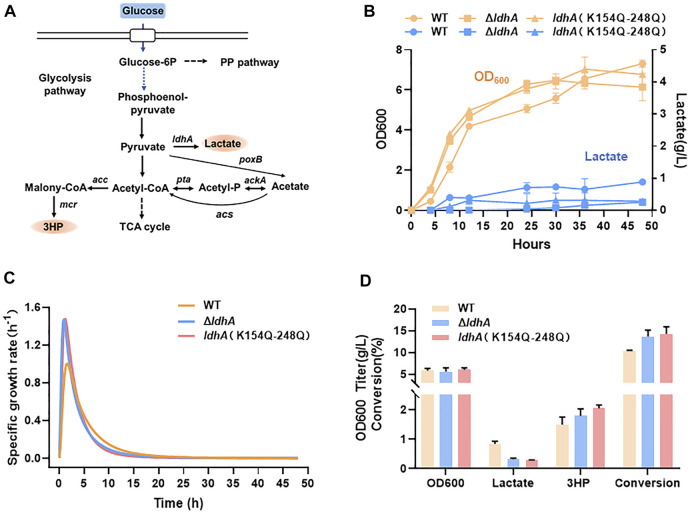
K154Q-K248Q mutant inhibits lactate accumulation and improves 3HP production. **(A)** The synthetic pathway of lactate and 3HP from glucose. **(B)** The cell growth and lactate accumulation of *E. coli* strains carrying wild-type LdhA and LdhA double mutant, and the *ldhA* deleted *E. coli* strain. The yellow line represents the cell growth, the blue line represents the lactate production. **(C)** The specific growth rate of these strains. **(D)** The biomass and metabolic profiles of these strains in 3HP fermentation.

Lactate is one of the most byproducts in microbial fermentation. In traditional methods, *ldhA* gene is usually deleted in engineered microorganism to overcome the lactate production and thus improve other chemicals production (Wu et al., 2009; Kumar et al., 2013; Lange et al., 2017). However, *ldhA* gene deletion may affect the replenishment of NAD and further inhibit cell growth. In this study, a double mutant K154Q-K248Q presented the lowest activity (Figure 1D). To explore the applications of this mutants in metabolic engineering, the chromosomal *ldhA* gene was *in situ* replaced by K154Q-K248Q variant and the cell growth and lactate synthesis were compared between the different gene modifications. In cell culture, the lactate concentrations were below 0.5 g/L in both *ldhA*-deleted strain (Δ*ldhA*) and *ldhA* (K154Q-K248Q) strain, significantly lower than that of the wild type (Figure 7B). The cell growth rates of these two mutants were higher in exponential phase, but they soon began to decline, especially in Δ*ldhA* strain (Figures 7B,C). Finally, the wild-type strain accumulated the highest biomass.

3-hydroxypropionate (3HP) is an attractive platform chemical for a wide range of industrial applications (Rathnasingh et al., 2009; Wang et al., 2012; Liu et al., 2013). We used 3HP as a case to study the application of LdhA acetylation in chemicals production *via* overcoming lactate accumulation. 3HP biosynthesis pathway from glucose was shown in Figure 7A. In 3HP fermentation, the Δ*ldhA* and *ldhA* (K154Q-K248Q) mutants kept a very low level of lactate and 3HP titers were 1.79 g/L and 2.05 g/L respectively, clearly higher than that produced by the wild-type strain of 1.57 g/L (Figure 7D). The 3HP conversion efficiencies of these mutants were improved by 1.33 and 1.38 times than that of the wild strain. More carbon flux may be assimilated to the pathway of 3HP biosynthesis due to the inhibition of byproduct synthesis. Therefore, LdhA acetylation modification can improve the 3HP biosynthesis *via* overcoming the lactate accumulation, and the regulatory effects are slightly better than the traditional method through gene knockout.

In conclusion, manipulating the lysine acetylation level could regulate the activity of related enzyme and affect the production of the desirable chemical. Lysine acetylation modification will be a promising regulatory strategy in microbial synthesis. How does control the acetylated level or sites? On one hand, we can identify the natural acetylated sites of proteins and regulate their acetylation level by mutation. On the other hand, a genetic code expansion approach can be used to incorporate acetyllysine directly into the selected positions. This approach utilizes an engineered pyrrolysyl-tRNA synthetase (Bryson et al., 2017) and a rationally evolved cognate tRNA pyl (Fan et al., 2015) to read through the TAG stop codon in the gene introduced by site-directed mutagenesis and incorporate the acetyllysine from medium to the selected sites. Venkat et al. (2017) studied the effects of lysine acetylation on enzyme activity of isocitrate dehydrogenase and malate dehydrogenase using this genetic code expansion approach (Venkat et al., 2018). This approach enables rational design of lysine acetylated sites and promotes the potential applications of lysine acetylation in microbial synthesis. Furthermore, as lactate has been proved to play an important role in pathogenesis of cancer and diabetes (Feng et al., 2018; Lin et al., 2022), our results may provide some important references for the treatment of these diseases.

## Conclusion

Protein lysine acetylation plays an important role in enzyme activity, metabolic flux distribution and other cellular physiology and metabolism processes, and its complex physiology effects and regulatory mechanism still remain unclear. This study systematically characterized the effects of lysine acetylated sites on *E. coli* LdhA and uncovered this regulatory mechanism. Lysine acetylation was also successfully used for regulating the lactate synthesis. Thus, our study established a paradigm for lysine acetylation to regulate lactate synthesis and proved that lysine acetylation can be a promising tool to improve the target production and conversion efficiency in metabolic engineering. Lysine acetylation is an evolutionarily conserved PTM from bacteria to higher animals and humans, and our results here may provide some important references for relevant research in other species.

## Data Availability

The original contributions presented in the study are included in the article/Supplementary Material, further inquiries can be directed to the corresponding authors.
