# Supplementary material for: Lysine acetylation of Escherichia coli lactate dehydrogenase regulates enzyme activity and lactate synthesis
Source: Front Bioeng Biotechnol. 2022 Aug 16;10:966062. doi: 10.3389/fbioe.2022.966062 (PMC9424733; doi:10.3389/fbioe.2022.966062)
Supplement: Supplementary file 1 [file DataSheet1.PDF]

Supplementary Information

Lysine acetylation of *Escherichia coli* lactate dehydrogenase regulates enzyme activity and lactate synthesis

Table S1 Primers used in this study

Name	Sequence
pETDuet1-*ldhA*-5’	CCGGGATCCGATGAAACTCGCCGTTTATAGC
pETDuet1-*ldhA*-3’	CCGGAGCTCTTAAACCAGTTCGTTCGGGC
pACYCDuet1-*patZ*-5’	CGCGGATCCGATGAGTCAGCGAGGACTGGAAGC
pACYCDuet1-*patZ*-3’	CCAAGCTTTCATGATTCCTCGCGCTGGGCAA
pACYCDuet1-*cobB*-5’	CGCGGATCCGATGCTGTCGCGTCGGGGTCATC
pACYCDuet1-*cobB*-3’	CCAAGCTTTCAGGCAATGCTTCCCGCTTTT
pET28a-*patZ*-5’	CGCGAATTCATGAGTCAGCGAGGACTGGA
pET28a-*patZ*-3’	CCCAAGCTTTCATGATTCCTCGCGCTGGG
pETDuet1-*ldhA*(K9R)-5’	AACTCGCCGTTTATAGCACA**CGT**CAGTACGACAAGAAGTACCT
pETDuet1-*ldhA*(K9R)-3’	AGGTACTTCTTGTCGTACTG**ACG**TGTGCTATAAACGGCGAGTT
pETDuet1-*ldhA*(K9Q)-5’	AACTCGCCGTTTATAGCACA**CAG**CAGTACGACAAGAAGTACCT
pETDuet1-*ldhA*(K9Q)-3’	AGGTACTTCTTGTCGTACTG**CTG**TGTGCTATAAACGGCGAGTT
pETDuet1-*ldhA*(K70R)-5’	AGCTGAAAAAGCACGGCGTT**CGT**TATATCGCCCTGCGCTGTGC
pETDuet1-*ldhA*(K70R)-3’	GCACAGCGCAGGGCGATATA**ACG**AACGCCGTGCTTTTTCAGCT
pETDuet1-*ldhA*(K70Q)-5’	AGCTGAAAAAGCACGGCGTT**CAG**TATATCGCCCTGCGCTGTGC
pETDuet1-*ldhA*(K70Q)-3’	GCACAGCGCAGGGCGATATA**CTG**AACGCCGTGCTTTTTCAGCT
pETDuet1-*ldhA*(K154R)-5’	CAGGCGTTATCGGTACCGGT**CGT**ATCGGTGTGGCGATGCTGCG
pETDuet1-*ldhA*(K154R)-3’	CGCAGCATCGCCACACCGAT**ACG**ACCGGTACCGATAACGCCTG

pETDuet1-*ldhA*(K154Q)-5’	CAGGCGTTATCGGTACCGGT**CAG**ATCGGTGTGGCGATGCTGCG
pETDuet1-*ldhA*(K154Q)-3’	CGCAGCATCGCCACACCGAT**CTG**ACCGGTACCGATAACGCCTG
pETDuet1-*ldhA*(K248R)-5’	AGGCAGCAATTGAAGCGCTG**CGT**AATCAGAAAATTGGTTCGTT
pETDuet1-*ldhA*(K248R)-3’	AACGAACCAATTTTCTGATT**ACG**CAGCGCTTCAATTGCTGCCT
pETDuet1-*ldhA*(K248Q)-5’	AGGCAGCAATTGAAGCGCTG**CAG**AATCAGAAAATTGGTTCGTT
pETDuet1-*ldhA*(K248Q)-3’	AACGAACCAATTTTCTGATT**CTG**CAGCGCTTCAATTGCTGCCT
pETDuet1-*ldhA*(D279E)-5’	CCAACGACGTGATCCAGGAT**GAA**GTATTCCGTCGCCTGTCTGC
pETDuet1-*ldhA*(D279E)-3’	GCAGACAGGCGACGGAATAC**TTC**ATCCTGGATCACGTCGTTGG
pETDuet1-*ldhA*(D279N)-5’	CCAACGACGTGATCCAGGAT**AAC**GTATTCCGTCGCCTGTCTGC
pETDuet1-*ldhA*(D279N)-3’	GCAGACAGGCGACGGAATAC**GTT**ATCCTGGATCACGTCGTTGG
pETDuet1-*ldhA*(E269D)-5’	ACGAACGCGATCTATTCTTT**GAT**GATAAATCCAACGACGTGAT
pETDuet1-*ldhA*(E269D)-3’	ATCACGTCGTTGGATTTATC**ATC**AAAGAATAGATCGCGTTCGT
pETDuet1-*ldhA*(E269N)-5’	ACGAACGCGATCTATTCTTT**AAC**GATAAATCCAACGACGTGAT
pETDuet1-*ldhA*(E269N)-3’	ATCACGTCGTTGGATTTATC**GTT**AAAGAATAGATCGCGTTCGT
ID-*pat*-5’	CCAATATTGTACTGCCGAGG
ID-*cobB*-5’	CTGACCTGGTCGTCATTGGT
ID-*pta*-5’	AAGACGCGAGCCGCCTGACTGCCTG
ID-*ackA*-5’	CATAAAACGGATCGCATAACGC
ID-*ldhA*-5’	CCACCAGTGACGGTTCCTGC
kan -In -3’	GGTGAGATGACAGGAGATCC
pRE112-up-5’	CCGTCTAGACAGTTGCTGGATATCAGAGG
pRE112-up-3’	GCTATAAACGGCGAGTTTCATAAGACTTTCTCCAGTGATGTTG
pRE112-*ldhA(K154Q-K248Q)*-5’	CAACATCACTGGAGAAAGTCTTATGAAACTCGCCGTTTATAGC
pRE112-*ldhA(K154Q-K248Q)*-3’	GGTTGCAGGGGAGCGGCAAGATTAAACCAGTTCGTTCGGGC

pRE112-dwon-5’	GCCCGAACGAACTGGTTTAATCTTGCCGCTCCCCTGCAACC
pRE112-down-3’	CCGGAGCTCGTCGATGTCCAGTAGTGGAG
ldhA -In -5’	GATCTGCCAACCCTGTTCTC

```
Pyrococcus        ---MKPKVLITRAIPENGIELLREH--FEVEVWEHEHEIPREVLLEKVKDVDALVTMLSE  55
Fusobacterium     MEKTKIIFFDIKDYDKEFFKKYEKDYNFEMTFLKVRLT---EETANLTKGYDVVCGFAND  57
Pseudomonas       ---MRILFFSTQAYDGESFQASNHRHGFELHFQQAHLQ---ADTAVLAQGFEVVCAFVND  54
Klebsiella        ---MKIAVYSTKQYDKKYLQHVNDTYGFELEFFDFLLT---AKTAKTANGCEAVCIFVND  54
Escherichia       ---MKLAVYSTKQYDKKYLQQVNESFGFELEFFDFLLT---EKTAKTANGCEAVCIFVND  54
Salmonella        ---MKLAVYSTKQYDKKYLQQVNEAFGFELEFFDFLLT---EKTAKTANGCEAVCIFVND  54
                      :  .   :    : ::  ..   **: . .             .:. :.:  : .:
Pyrococcus        KIDREVFDA--APRLRIVANYAVGYDNIDIEEATKRGIYVTNTPDVLTDATADLAWALLL  113
Fusobacterium     NINKETIDIMAKNGIKLLAMRCAGFNNVSLKDIH-NRFKVVRVPAYSPHAIAEYTVGLIL  116
Pseudomonas       DLSRPVLERLAAGGTRLVALRSAGYNHVDLAAAEALGLPVVHVPAYSPHAVAEHAVGLIL  114
Klebsiella        DGSRPVLEELKAHGVKYIALRCAGFNNVDLEAAKELGLRVVRVPAYSPEAVAEHAIGMMM  114
Escherichia       DGSRPVLEELKKHGVKYIALRCAGFNNVDLDAAKELGLKVVRVPAYDPEAVAEHAIGMMM  114
Salmonella        DGSRPVLEELKKHGVKYIALRCAGFNNVDLDAAKELGLQVVRVPAYSPEAVAEHAIGMMM  114
                  . .: .::       : :*  ..*::::.:       : *...*    .* *: : .:::
Pyrococcus        AAARHVVKGDKFVRSGEWKRRGIAWHPKMFLGYDVYGKTIGIVGFGRIGQAIAKRAKGFG  173
Fusobacterium     AVNRKIHKAYVRTREGNFSING-------LMGFDLDGKTVGIIGTGKIGQILIKILKGFD  169
Pseudomonas       TLNRRLHRAYNRTREGDFSLHG-------LTGFDLHGKRVGVIGTGQIGETFARIMAGFG  167
Klebsiella        SLNRRIHRAYQRTRDANFSLEG-------LTGFTMYGKTAGVIGTGKIGVAMLRILKGFG  167
Escherichia       TLNRRIHRAYQRTRDANFSLEG-------LTGFTMYGKTAGVIGTGKIGVAMLRILKGFG  167
Salmonella        TLNRRIHRAYQRTRDANFSLEG-------LTGFTMHGKTAGVIGTGKIGVAALRILKGFG  167
                  :  *:: :.   .*..::. .*       : *: : **  *::* *:**    :   **.
Pyrococcus        MRILYTARSRKPEAEKELGAEFKPLEELLRESDFVVLAVPLTKETYHMINEERLRLMKPT  233
Fusobacterium     MKVIAYDLFPNQKVADELGFEYVSLDELYAKSDIISLNCPLTKDTKYMINRRSMLKMKDG  229
Pseudomonas       CELLAYDPYPNPR-IQALGGRYLALDALLAESDIVSLHCPLTADTRHLIDAQRLATMKPG  226
Klebsiella        MRLLAFDPYPSAA-ALELGVEYVDLATLYKESDVISLHCPLTDENYHLLNREAFDQMKDG  226
Escherichia       MRLLAFDPYPSAA-ALELGVEYVDLPTLFSESDVISLHCPLTPENYHLLNEAAFEQMKNG  226
Salmonella        MRLLAFDPYPSTT-ALDLGVEYVDLQTLFAESDVISLHCPLTPENYHLLNHAAFDQMKNG  226
                   .::      .      ** .:  *  *  :**.: *  *** :. ::::   :  **
Pyrococcus        AVLVNVARGKVVDTKALIRALKEGWIAAAGLDVFEEEPYYD-------------EELFA  279
Fusobacterium     VILVNTGRGQLIDSADLVEALKDKKIGAVALDVYEEEENYFFEDKSNQVIEDDILGRLLS  289
Pseudomonas       AMLINTGRGALVNAAALIEALKSGQLGYLGLDVYEEEADIFFEDRSDQPLQDDVLARLLS  286
Klebsiella        VMVINTSRGALIDSQAAIDALKHQKIGALGLDVYENERDLFFEDKSNDVIQDDVFRRLSA  286
Escherichia       VMIVNTSRGALIDSQAAIEALKNQKIGSLGMDVYENERDLFFEDKSNDVIQDDVFRRLSA  286
Salmonella        VMVINTSRGALIDSQAAIEALKNQKIGSLGMDVYENERDLFFEDKSNDVIQDDVFRRLSA  286
                  .:::*..** ::::   : ***   :.  .:**:*:*                   .* :
Pyrococcus        LDNVVLTPHIGSATFGAREGMAELVAKNLIAFKNGEVPPTLVNREVLKVRRPGF------  333
Fusobacterium     FYNVLITSHQAYFTKEAVDAITVTTLNNIRDFIEGKPLVNEVLQN---------------  334
Pseudomonas       FPNVVVTAHQAFLTREALAAIADTTLDNIAAWQDGTPRNRV--RA---------------  329
Klebsiella        CHNVLFTGHQAFLTAEALISISETTLGNLQQVANGETCPNAIV-----------------  329
Escherichia       CHNVLFTGHQAFLTAEALTSISQTTLQNLSNLEKGETCPNELVSALMNELLAENTERICA  346
Salmonella        CHNVLFTGHQAFLTAEALISISETTLQNLSQLEKGEACPNALF-----------------  329
                    **:.* * .  *  *  .::  .  *:    .*
```

Fig. S1. Sequence alignment of LdhA in bacteria
